# Loss to follow-up before and after initiation of antiretroviral therapy in HIV facilities in Lilongwe, Malawi

**DOI:** 10.1371/journal.pone.0188488

**Published:** 2018-01-26

**Authors:** Hannock Tweya, Ikwo Kitefre Oboho, Salem T. Gugsa, Sam Phiri, Ethel Rambiki, Rebecca Banda, Johnbosco Mwafilaso, Chimango Munthali, Sundeep Gupta, Moses Bateganya, Alice Maida

**Affiliations:** 1 The International Union Against Tuberculosis and Lung Disease, Paris, France; 2 Lighthouse Trust, Lilongwe, Malawi; 3 Division of Global HIV and Tuberculosis (DGHT), Center for Global Health, Centers for Disease Control and Prevention (CDC), Atlanta, Georgia, United States of America; 4 International Training and Education Center for Health (I-TECH), Lilongwe, Malawi; 5 Department of Public Health, School of Public Health and Family Medicine, College of Medicine, University of Malawi, Lilongwe, Malawi; 6 Department of Medicine, School of Medicine, University of North Carolina Chapel Hill, Chapel Hill, NC, United States of America; 7 Bwaila Hospital, Ministry of Health, Lilongwe, Malawi; 8 Baobab Health Trust, Lilongwe, Malawi; 9 Division of Global HIV and Tuberculosis (DGHT), Center for Global Health, Centers for Disease Control and Prevention (CDC), Lilongwe, Malawi; NPMS-HHC CIC / LSH&TM, UNITED KINGDOM

## Abstract

**Introduction:**

Although several studies have explored factors associated with loss to follow-up (LTFU) from HIV care, there remains a gap in understanding how these factors vary by setting, volume of patient and patients’ demographic and clinical characteristics. We determined rates and factors associated with LTFU in HIV care Lilongwe, Malawi.

**Methods:**

We conducted a retrospective cohort study of HIV-infected individuals aged 15 years or older at the time of registration for HIV care in 12 ART facilities, between April 2012 and March 2013. HIV-positive individuals who had not started ART (pre-ART patients) were clinically assessed to determine ART eligibility at registration and during clinic follow-up visits. ART-eligible patients were initiated on triple antiretroviral combination. Study data were abstracted from patients’ cards, facility ART registers or electronic medical record system from the date of registration for HIV care to a maximum follow-up period of 24 months. Descriptive statistics were undertaken to summarize characteristics of the study patients. Separate univariable and multivariable poisson regression models were used to explore factors associated with LTFU in pre-ART and ART care.

**Results:**

A total of 10,812 HIV-infected individuals registered for HIV care. Of these patients, 1,907 (18%) and 8,905 (82%) enrolled in pre-ART and ART care, respectively. Of the 1,907 pre-ART patients, 490 (26%) subsequently initiated ART and were included in both the pre-ART and ART analyses. The LTFU rates among patients in pre-ART and ART care were 48 and 26 per 100 person-years, respectively. Of the 9,105 ART patients with reasons for starting ART, 2,451 (27%) were initiated on ART because of pregnancy or breastfeeding (Option B+) status. Multivariable analysis showed that being ≥35 years and female were associated with decreased risk of LTFU in the pre-ART and ART phases of HIV care. However, being in WHO clinical stage 3 (adjusted risk ratio (aRR) 1.35, 95% confidence interval (CI): 1.20–1.51) and stage 4 (aRR 1.87, 95% CI: 1.62–2.18), body mass index ≤ 18.4 (aRR 1.24, 95% CI: 1.11–1.39) at ART initiation, poor adherence to clinic appointments (aRR 4.55, 95% CI: 4.16–4.97) and receiving HIV care in rural facilities (aRR 2.32, 95% CI: 1.94–2.87) were associated with increased risk of LTFU among ART patients. Being re-initiated on ART once (aRR 0.20, 95% CI: 0.17–0.22), more than once (aRR 0.06, 95% CI: 0.05–0.07), and being enrolled at a low-volume facility (aRR 0.25, 95% CI: 0.20–0.30) were associated with decreased risk of LTFU from ART care.

**Conclusion:**

A sizeable proportion of ART LTFU occurred among women enrolled during pregnancy or breast-feeding. Non- compliance to clinic and receiving ART in a rural facility or high-volume facility were associated with increased risk of LTFU from ART care. Developing effective interventions that target high-risk subgroups and contexts may help reduce LTFU from HIV care.

## Introduction

Increased access to antiretroviral therapy (ART) in resource-limited settings is an unprecedented success story for HIV programs. As of June 2015, 15.8 million people received ART globally, the majority, 10.7 million in sub-Saharan Africa (SSA)[1], a tremendous increase from just over 0.8 million people in 2005 [2]. However, these successes are being challenged by high rates of loss to follow-up (LTFU) [3].

LTFU from HIV care is a major public health concern. LTFU increases the risk of drug resistance and treatment failure in cases where death is not the reason for LTFU. Previous studies showed that patients with treatment interruptions are more likely to develop treatment failure [4,5], viral rebound, and clinically significant drug resistance [4]. At the national program and clinic levels, not accounting for LTFU may bias retention estimates and misdirect resources when planning and budgeting for HIV care [6].

Factors and reasons for LTFU at patient and system levels include advanced HIV disease, older age, male gender, longer distance to clinics in patients not on ART, unregistered transfers out and undocumented deaths in patients on ART [6–9]. Factors associated with LTFU remain complex. Comparing facility characteristics in urban and rural HIV with varying patient volumes may inform the design of intervention that optimize retention.

Effective early patient tracing may reduce LTFU and improve retention. However, tracing all patients who have missed appointments and those LTFU can be resource and time-intensive. As such, the Malawi guidelines recommend prioritizing tracing patients that miss ART appointment or those eligible for ART [10]. Despite this guidance, implementation is challenged by health systems constraints including chronic human resource shortages and limited health infrastructure; as a result, facility- and community-level interventions to improve retention differs widely across the country.

Optimizing retention by mitigating the varying risk factors for LTFU may greatly impact Malawi’s trajectory towards epidemic control. Retention interventions may be optimised in terms of person, place, and time by identifying individual-level or context specific risk factors that may predispose a patient to be LTFU. This paper describes LTFU and associated factors in pre-ART and ART patients at 12 ART facilities, prior to the implementation of a quality improvement (QI) collaborative aimed at optimizing retention in care in Lilongwe district, Malawi.

## Methods

### Setting

Data used for this study was obtained from five rural and seven urban ART facilities in Lilongwe District, Malawi. All six high-volume ART facilities, with more than 400 new ART patients per year, used an electronic medical records system (EMRs) for patient management and the remaining low-volume facilities used paper-based systems. Five high-volume facilities were located in urban setting while one high-volume facility was in rural setting. HIV-infected adults were registered for HIV care in the EMRs or paper patient cards. During the observation period, ART eligibility was defined in accordance with the national guidelines: World Health organisation (WHO) clinical stage 3 or 4 or having a CD4 count of ≤ 350 cells/μl. Pregnant and breastfeeding women were also considered eligible irrespective of their CD4 count or WHO clinical stage as Malawi had adopted Option B+ for prevention of mother-to-child transmission (PMTCT) of HIV. Pre-ART patients, HIV-positive individuals who had not started ART (pre-ART patients), were clinically assessed based on WHO clinical criteria every 2 months to determine ART eligibility. For those not eligible based on WHO clinical stage, CD4 was repeated every 6 months when feasible. Therefore, CD4 data were generally available for patients in WHO stage 1 or 2. At each visit, pre-ART and ART patients received co-trimoxazole as prophylaxis to prevent opportunistic infections. ART-eligible patients were initiated on triple antiretroviral (ARV) combination: Tenofovir, Lamivudine, and Efavirenz and routine follow-up visits were scheduled monthly or every two months. Based on the data collection system, subsequent ART clinic appointments were either calculated manually or electronically based on the ARV regimen and prescribed schedule, number of tablets dispensed, and the number of tablets remaining from the previous dispensing visit. ART programme outcomes (alive and on ART, stopped ART, or transferred-out) were updated on the patient card or in EMRs at each clinic visit or retrospectively. Stopping ART, transferring care or death were also updated retrospectively in facilities with patient tracing systems.

### Study design and population

This retrospective cohort study used routine clinic data collected from HIV-infected individuals who were 15 years of age or older at the time of registration for HIV care in the ART facilities, between April 2012 and March 2013.

### Variable definitions

Pre-ART patients comprised of two categories of patients relative to ART; patients who were not eligible for ART at baseline and those eligible but never started ART for at least three months despite attending clinic consultations. The primary outcome variable was LTFU defined as failure to come to the clinic for at least 60 days from the patient’s appointment date. Patients were classified as (i) “stopped treatment” when they stopped ART during the follow-up period including medical reasons; (ii) “transferred-out” if the patient had a formally recorded transfer to another clinic, (iii) “dead” if the patient died from any cause. Other variables of interest were adherence to clinic appointments and ART re-initiation. Adherence to clinic appointments was defined as having at least 80% of the ART visits *before* 7 days after a scheduled appointment. Re-initiation was defined as restarting ART treatment after stopping ART for at least 60 days.

### Data collection and statistical analysis

Data were abstracted from individual patient cards, facility ART registers or EMRs from the date a patient registered at an HIV facility up to a maximum follow-up period of 24 months. Patients who initially registered for pre-ART services and later started ART had their data abstracted for pre-ART and for ART follow-up periods. A time-to-event analysis was conducted for pre-ART and ART times, separately. For person-time calculation, the analysis period began when patients enrolled in pre-ART care or initiated ART at the facility. Observation of ‘time at risk’ for LTFU during pre-ART care ended either at the time of an outcome (started ART, LTFU, transfer-out, or death) or at 24 months after pre-ART registration. For ART patients, observation of ‘time at risk’ of LTFU ended either at the time of an outcome (stopped ART, LTFU, transferred-out, or death) or at 24 months after ART registration. Separate Poisson regression models were used to explore factors associated with LTFU in pre-ART and ART care. A null hypothesis of no within-facility clustering, versus the alternative of some within-facility clustering were tested (p = 0.065). All the analysis did not account for clustering at facility level because within facility variability was not significant. The models were adjusted for age and sex, *a priori*. Model results were shown as risk ratios (RR) and adjusted RR (aRR) with 95% confidence intervals (CI).

### Ethics approval

The study was approved by the Malawi National Health Science Research Committee in Lilongwe, Malawi and by the office of the Associate Director for Science of the U.S. Centers for Disease Control and Prevention in Atlanta, Georgia.

## Results

A total of 10,812 HIV-positive individuals were enrolled into HIV care at the 12 facilities between April 2012 and March 2013; of these patients, 1,907 (18%) registered for pre-ART care and 8,905 (82%) for ART care. Of the 1,907 pre-ART patients, 490 (26%) subsequently started ART and were included in both the pre-ART and ART analyses, resulting in a total of 9397 ART patients. The majority of patients were enrolled in six high-volume facilities; 1,681 (88%) of pre-ART patients and 8,390 (89%) of ART patients. On average, low-volume facilities enrolled 170 (range 99−221) patients per year while large volume facilities enrolled 1330 (range 403−4133) patients per year. There were no significant differences in demographic characteristics between patients enrolled at low and high volume facilities.

### Pre-ART patients

Among the pre-ART patients, 1,098 (58%) were female (**Table 1**). Median age at enrolment in pre-ART care was 31 years (interquartile range (IQR): 26−38). Median CD4 count was 409 cells/μl (IQR: 290–555); men had lower median CD4 counts compared to women (374 vs 441 cells/μl, p <0.001).

**Table 1:** Baseline characteristics of HIV-infected persons who registered for pre-ART and ART care between April 2012 and March 2013 in Lilongwe, Malawi.

Characteristics	Pre-ART					ART				
	Total	Low-volume load facilities (n = 226)		High-volume load facilities * (n = 1681)		Total	Low-volume load facilities (n = 1107)		High-volume load facilities* (n = 8380)	
Gender										
Male	809 (42%)	97	43%	712	42%	3162 (34%)	343	34%	2819	34%
Female	1098 (58%)	129	57%	969	58%	6213 (66%)	653	66%	5560	66%
*Unknown*						22 (<1%)	21	2%	1	<1%
Age										
15–24	361 (19%)	27	12%	334	20%	1582 (17%)	117	12%	1465	17%
25–34	865 (45%)	97	43%	768	46%	4189 (45%)	407	43%	3782	45%
35–44	457(24%)	64	29%	393	23%	2426 (26%)	275	29%	2151	26%
≥45	220 (12%)	35	16%	185	11%	1131 (12%)	150	16%	981	12%
*Unknown*	4 (<1%)	3	1%	1	<1%	69 (<1%)	68	7%	1	<1%
BMI category										
<18.5	157 (9%)	17	13%	140	9%	1356 (15%)	129	19%	1227	15%
18.5–24.9	1238 (70%)	95	70%	1143	70%	5689 (64%)	423	62%	5266	64%
≥25.0	367 (21%)	24	18%	343	21%	1819 (21%)	135	20%	1684	21%
*Unknown*	145 (8%)	90	3%	55	40%	533 (6%)	330	32%	203	2%
CD4 cell count at registration										
<350	376 (34%)	17	15%	359	37%	2375 (92%)	242	92%	2133	92%
350–500	363 (33%)	48	42%	315	32%	175 (7%)	11	4%	164	7%
>500	354 (32%)	50	43%	304	31%	32 (1%)	9	3%	23	1%
*Unknown*	814 (43%)	111	49%	703	42%	6815 (73%)	755	74%	6060	72%
WHO clinical stage										
1 or 2	1352 (83%)	101	81%	1251	83%	3927 (50%)	561	59%	3366	48%
3	222 (14%)	23	19%	199	13%	3149 (40%)	302	32%	2847	41%
4	61 (3%)	0	0%	61	4%	828 (10%)	95	10%	733	11%
*Unknown*	272 (14%)	102	45%	170	10%	1493 (16%)	59	6%	1434	17%

*High-volume load facilities enrolled at least 400 ART patients per year

BMI: body mass index; ART: antiretroviral therapy, unknown category is excluded in the percentage calculations

### ART patients

Of the 9,397 ART patients, 6,213 (66%) were female **(Table 1).** Median age at ART initiation was 32 years (IQR: 27−38) and the majority (45%) of patients were aged 25−34 years. Among those with documented WHO clinical stage, 50% had WHO stage 3 or 4 conditions. Among ART patients with documented CD4 counts, women had higher median CD4 counts at ART initiation than men, 241 cells/μl and 186 cells/μl, respectively. Of all patients who initiated ART, 2,451 (26%) were pregnant or breastfeeding and started on ART for PMTCT and 8,380 (89%) were enrolled at high-volume facilities.

### Pre-ART and ART program outcomes by facility size

Median follow-up time for the 1,907 pre-ART patients was 8.5 (2.4−23) months. Overall rate of LTFU was 48 (95% CI, 45−52) per 100 person-years. Low-volume facilities did not document any LTFU patients at 12 or 24 months of follow-up (**Table 2**). In contrast, among 1,681 pre-ART patients enrolled for HIV care at high-volume facilities, 47% were LTFU after 12 months of follow-up; at 24 months, 54% patients were LTFU. There were no patients reported as transferred-out or dead both at 12 and 24 months of follow-up in pre-ART care at high-volume facilities.

**Table 2:** ART program outcomes by time in HIV care among HIV-infected persons who registered for pre-ART and ART care between April 2012 and March 2013 in Lilongwe, Malawi.

	Low-volume load facilities				High-volume load facilities*			
	0–12 months		0–24 months		0–12 months		0–24 months	
**Pre-ART**								
Total registered	226		226		1681		1681	
Follow-up outcome								
pre-ART follow-up	184	81%	158	70%	637	38%	351	21%
Started ART	39	17%	64	28%	253	15%	426	25%
LTFU^¥^	0	0%	0	0%	791	47%	904	54%
Transferred out	2	<1%	3	<1%	0	0%	0	0%
Dead	1	<1%	1	<1%	0	0%	0	0%
**ART**								
Total registered	1017		1017		8380		8380	
ART outcome^β^								
Alive and on ART	740	75%	675	69%	5097	61%	4174	50%
LTFU	207	21%	258	26%	2071	25%	2699	32%
Stopped ART	1	0%	1	<1%	7	<1%	12	<1%
Transferred out	8	1%	11	1%	1178	14%	1467	18%
Dead	21	2%	32	3%	18	<1%	19	<1%

*High-volume load facilities enrolled at least 400 ART patients per year

^¥^LTFU: Loss to follow-up

^β^40 patients had no ART outcomes in paper-based facilities and 9 had no ART outcomes in EMR-based facilities

Of the 9,397 ART patients, 49 had missing outcomes and 9,348 ART patients contributed 11,527 person-years. The overall rate of LTFU in ART patients was 26 (95% CI 25–27) per 100 person-years and the median follow-up was 20.9 (IQR 3–23) months. ART outcomes were not documented in 49 patients: 40 enrolled in low-volume facilities and 9 in high-volume facilities. At 12 months post-ART initiation, more patients were reported to be alive and on ART in low-volume facilities than in high-volume facilities (p<0.001) and the difference was still significant at 24 months (Table 2).

### Factors associated with loss to follow-up from pre-ART and ART care

In univariable analysis of pre-ART patients, having a body mass index (BMI) < 18.4, baseline WHO stage 3 and stage 4 were associated with an increased risk of LTFU (Table 3). Being female, age 35−44 years, or age ≥45 years and having a baseline CD4 count of >350 cells/μl were associated with a decreased risk of LTFU. These factors remained significantly associated with LTFU after adjusting for potential confounders in the multivariable analysis.

**Table 3:** Univariable and multivariable model of characteristics associated with loss to follow-up from pre-ART care in Lilongwe, Malawi.

Characteristics	n	Unadjusted RR (95% CI)		P-value	Adjusted RR (95% CI)		P-value
Gender				<0.001			<0.001
Female	1098	0.75	(0.66–0.86)		0.74	(0.64–0.87)	
Male	809	1.00			1.00		
Age				<0.001			<0.001
15–24	361	1.11	(0.94–1.31)		1.13	(0.95–1.36)	
25–34	865	1.00			1.00		
35–44	457	0.79	(0.66–0.94)		0.72	(0.59–0.87)	
45+	220	0.77	(0.61–0.96)		0.73	(0.57–0.94)	
BMI category				0.042			0.113
<18.5	157	1.26	(1.01–1.57)		1.20	(0.94–1.54)	
18.5–24.9	1238	1.00			1.00		
25.0+	367	0.85	(0.72–1.01)		0.90	(0.75–1.09)	
CD4 count at registration				<0.001			
<350	376	1.00					
350–500	363	0.43	(0.35–0.54)		-	-	
>500	354	0.56	(0.46–0.69)		-	-	
WHO clinical stage				0.001			0.001
1 or 2	1352	1.00			1.00		
3	222	1.35	(1.11–1.64)		1.31	(1.07–1.60)	
4	61	1.68	(1.18–2.37)		1.60	(1.13–2.27)	

RR: risk ratio; CI: confidence interval; adjusted models were based on available data only; CD4 was excluded in the multivariable analysis because data were available in 57% only

In univariable analysis of ART patients, being female, younger age 15−24 years, BMI <18.5, baseline WHO stages 3 and 4 were associated with increased risk of LTFU (Table 4). Women who started ART based on Option B+ criteria had a higher risk of LTFU than patients who started ART based on CD4 count. The risk of LTFU was higher among patients who were not adherent to scheduled clinic appointments compared to those who were adherent. Predictors of decreased risk of LTFU included age 35years and above, being re-initiated on ART once, being enrolled at a low-volume facility and receiving care from an urban facility.

**Table 4:** Univariable and multivariable model of characteristics associated with loss to follow-up from ART care in Lilongwe, Malawi.

Characteristics	n^Ω^	Unadjusted RR(95% CI)		P-value	Adjusted RR(95% CI)		P-value
Gender				<0.001			<0.001
Female	6190	1.27	(1.18–1.38)		0.84	(0.76–0.93)	
Male	3139	1.00			1.00		
Age				<0.001			<0.001
15–24	1577	1.42	(1.29–1.55)		1.35	(1.21–1.49)	
25–34	4173	1.00			1.00		
35–44	2411	0.65	(0.59–0.72)		0.70	(0.63–0.78)	
≥45	1122	0.67	(0.59–0.77)		0.75	(0.65–0.88)	
BMI category				<0.001			<0.001
<18.5	1354	1.22	(1.10–1.36)		1.24	(1.11–1.39)	
18.5–24.9	5673	1.00			1.17		
≥25.0	1,816	0.94	(0.85–1.03)		0.90	(0.82–1.00)	
Reason for starting ART				<0.001			
CD4 below threshold	2692	1.00			1.00		<0.001
WHO 3	3140	1.46	(1.32–1.62)		1.34	(1.20–1.51)	
WHO 4	826	1.98	(1.72–2.26)		1.87	(1.62–2.18)	
Pregnant/breastfeeding	2447	2.51	(2.27–2.76)		1.77	(1.57–1.99)	
Adherence to clinic appointment ^€^				<0.001			<0.001
Yes	4625	1.00			1.00		
No	4723	2.71	(2.51–2.93)		4.55	(4.16–4.97)	
ART re-initiation				<0.001			<0.001
0	6287	1.00			1.00		
1	1825	0.42	(0.38–0.47)		0.20	(0.17–0.22)	
≥2	887	0.19	(0.15–0.23)		0.06	(0.05–0.07)	
Facility location				<0.025			<0.001
Rural	981	0.88	(0.78–0.99)		2.32	(1.94–2.77)	
Urban	8367	1.00			1.00		
Facility patient load*				<0.001			<0.001
Low-volume	977	0.65	(0.57–0.74)		0.25	(0.20–0.30)	
High-volume	8371	1.00			1.00		

^**Ω**^Included only patients with follow-up time; RR: risk ratio, CI: confidence interval

** High-volume load facilities enrolled at least 400 ART patients per year

^€^Compliance; at least 80% of visits happened before 7 days after the scheduled clinic appointment date; ART re-initiation; starting ART after failure to come to the clinic for at least 60 days from the date the patient was expected to return to the clinic: Some patients had no data for age (46(<1%), reason for starting ART (224(2%)) and ART reinitiation (349(4%)

In the multivariable analysis of ART patients, the association between baseline age, BMI, reason for starting ART, adherence to clinic appointments, ART re-initiation and patient volume facility and LTFU remained significant; the risk of LTFU among patients who were not adherent to clinic appointments was more pronounced (Table 4). After adjusting for other factors, the associations between gender and location of the facility with LTFU were reversed; being female, and receiving ART at a rural facility associated with an increased risk of LTFU. Adherence to scheduled clinic appointments was poor if the reason for starting ART was pregnancy, breastfeeding, WHO stages 3 or 4 unlike in cases where treatment was started solely based on a CD4 count of less than 350 cells/μl.

## Discussion

This study describes LTFU from pre-ART and ART care at urban and rural HIV facilities with low and high patient volumes. Overall LTFU was 48% and 26% per year in the pre-ART and ART cohorts, respectively. Among the pre-ART and ART cohorts, being 35 years of age or older and female were associated with reduced risk of LTFU. Advanced WHO clinical stage, low BMI at ART initiation, poor adherence to clinic appointments and receiving HIV care in rural facilities on the other hand were associated with increased risk of LTFU among ART patients. Re-initiation of ART and receiving ART care at low-volume facilities on the other hand were associated with decreased risk of LTFU.

Our findings highlight critical LTFU issues in pre-ART and ART cohorts. In the pre-ART cohort, we observed an overall LTFU of 48% per year, which falls within the range of 23–88% reported in a systematic review from SSA[11]. However, LTFU in our study was higher than previously reported among pre-ART patients in Malawi possibly because of different definitions of pre-ART patients[12][13]. Other studies used a limited definition of pre-ART patients i.e. including only those in WHO stage 1 and 2 who were not eligible to start ART. In this study, we defined pre-ART patients more broadly as all persons newly enrolled in HIV care who had not started ART three months post-clinic registration, regardless of their clinical stage or CD4 count. All low-volume facilities, using paper-based data management systems, did not document LTFU status in pre-ART patient card, despite the national guidelines recommendation to document and report all persons who have missed their appointments by 60 days as LTFU. High-volume facilities, on the other hand, have an in-built EMR feature that automatically assigns LTFU status while paper-based systems require that staff review and update patient cards for patients who are LTFU. Documentation of some data elements was suboptimal at low-volume facilities compared to high-volume facilities. For example, BMI and WHO clinical stage were not documented for 40% and 45% of the pre-ART patients’ records respectively versus 3% and 10% in high-volume facilities with EMR. In addition, the LTFU rates were almost twice as high in the pre-ART cohort compared to ART cohort. This may be because of less attention being given to towards interventions that reduce LTFU in pre-ART prior to the test-and-treat era.

Since July 2016, Malawi implemented the test-and-treat approach for ART initiation[14]. Given referral challenges, special clinical considerations, personal preferences and care provider’s recommendations, not all newly identified HIV positive individuals will initiate ART on the same day of testing or within seven days as recommended by Malawi guidelines. HIV-infected individuals that have not been initiated on ART on the same day of testing may need to be prioritized by enhancing measures that encourage utilization of HIV care services and active patient tracing to prevent high LTFU rates.

Among patients on ART, we observed LTFU of 26% per year, higher in high-volume facilities (27%) compared to low-volume facilities (17%). We acknowledge that some of the ART patients may have continued ART elsewhere without a formal transfer-out note, died or simply stopped ART as previous research in Malawi showed [6]. However, the rate of LTFU was still higher than previously reported in the Malawi national ART program where it was 9% per year prior to the implementation of Option B+ [9]. Previous studies have shown high LTFU among women who initiate ART while pregnant or breastfeeding [15–17]. A quarter of the ART patients in this study started ART based on Option B+ criteria and contributed to 39% of the all LTFU patients were LTFU from Option B+. We previously conducted a study where we noted that 47% of pregnant women LTFU received ART only once from a health care provider and missed subsequent appointments, indicating that they may never have started ARVs[16]. Same day ART initiation for pregnant and breastfeeding women may result in inadequate patient preparation, missed opportunities for disclosure, and social support, all of which can contribute to LTFU. Being asymptomatic may contribute to unwillingness to comply with scheduled appointment dates among pregnant and breastfeeding women because they feel healthy and do not perceive the need for medications to sustain their health. Comprehensive interventions to reduce LTFU need to specifically target pregnant and breastfeeding women who start ART for life.

We also observed that high-volume facilities reported higher LTFU rates compared to low-volume facilities. One possible explanation for this may be that high patient-to-provider ratios limit individualized patient education, patient preparation before ART initiation, and ongoing counselling once individuals are on lifelong therapy. Models of HIV service delivery that reduce congestion at health facilities including community-based ART distribution systems, multi-month ART appointments, utilization of other cadres and/or task shifting for adherence counseling, and providing larger quantities of ARVs per visit for stable patients. Similar to previous studies of pre-ART cohorts, we found that being female, older than 35 years, and having a high CD4 count were associated with a lower risk of LTFU [11][18]. On the other hand, low BMI and the presence of WHO stages 3 or 4 defining illnesses at enrollment was associated with a high risk of LTFU. Some of the patients with WHO stages 3 or 4 may have experienced unreported deaths [18]. In cases where CD4 criteria was used for ART eligibility, low CD4 were found to be at risk for LTFU most likely for reason similar to those with WHO stages 3 or 4.

Among those who started ART, patients were less likely to be LTFU again if they returned to care and re-initiated ART. The decreased LTFU after re-initiation of care may also be attributed to the stable lifestyles of individuals in regards to living with HIV and being in care, to the counseling they received upon return to care, or the experience of negative outcomes such as opportunistic infections and weight loss encountered when care was interrupted. We also noted a high risk of LTFU among younger patients, women, those in urban setting, and those who received care at high-volume facilities. In the multivariable analysis, being female was associated with a lower risk of LTFU after adjusting for confounding factors such as age, reason for starting treatment and compliance to scheduled appointment dates. Younger women, those who started ART due to pregnancy or breastfeeding, and those who had never been reinitiated on treatment were more likely to be LTFU, while those reinitiated on treatment at least once were less likely to be LTFU. These factors may be contributors to LTFU in countries that have adopted a universal Test and Treat strategy for pregnant and breastfeeding women. HIV programs that use Option B and B+ strategies will need to employ specific interventions, such as active tracing of patients that miss their appointments, prioritization of pregnant and breastfeeding women to optimize retention to care. On the other hand, receiving care from low-volume facilities where patient-to-provider ratios may be lower and may allow for more patient-centered care was associated with lower risk of LTFU–thereby contributing to better adherence to scheduled clinic appointments. An additional factor that negatively impacted adherence to clinic visits was receiving care in a rural facility. ART reinitiating also negatively influenced the risk for LTFU among people receiving ART in a rural facility. Non-adherence to clinic visits has been reported to be independently associated with all-cause mortality [19].

In Malawi, some high-volume facilities are piloting Demographic Data Exchange (DDE) systems that generate a unique national identification (ID). The ID enable tracking of unique HIV positive individuals at different points across the HIV care continuum. Such approaches facilitate tracking of ART patients who transfer care without a formal clinical documentation. The DDE technology can be utilized to identify patients who access ARVs at another facility and determine patients who transfer or receive ad-hoc care without a formal documentation. Expanding such systems however will also need to consider how such technology can be mirrored in facilities that use paper-based records. Additionally, such systems will need to be implemented in parallel with the development and implementations of privacy laws and guidelines on protecting the confidentiality and security of personal health information.

Our study is subject to some limitations that should be taken into account when interpreting the findings. First, some patients might have been classified as LTFU simply because their true status was not known by the facility where they first sought care. We cannot therefore rule out the possibility that some patients transferred their care elsewhere or may have died. Second, we may have underestimated the true incidence of LTFU due to the incomplete documentation at paper-based facilities. Third, it is possible that some of the patients classified as LTFU may have died resulting in an overestimation of LTFU. Fourth, pre-ART and ART data were not available for some patients in paper-based facilities. Since the models were based on variables with variable data, this might have led to selection bias in our estimates. Lastly, we could not explore the effect of patient volume and location of the facility on pre-ART LTFU because of the small numbers of LTFU patients. Despite these limitations, our study findings shed light on additional factors related to LTFU such as adherence to clinic appointment, patient volume, re-initiation of ART and the effect of Option B+ on the overall ART cohort.

In summary, higher rates of LTFU were observed in pre-ART care when compared with ART care. Women who started ART because of Option B+ contributed significantly to LTFU. Non-adherence to clinic appointments, and receiving HIV care in a high-volume facility or rural facility were associated with increased risk of LTFU. Data incompleteness within paper-based data systems.is a challenge that calls for standardized operating systems or EMRs that monitor and easily flag LTFU patients in pre-ART and ART care; this may promote actions that reduce LTFU. Interventions that target high risk sub-groups and contexts may also prevent LTFU.
